# Cycling is the most important predictive split discipline in professional Ironman® 70.3 triathletes

**DOI:** 10.3389/fspor.2024.1214929

**Published:** 2024-02-08

**Authors:** Katja Weiss, David Valero, Marilia Santos Andrade, Elias Villiger, Mabliny Thuany, Beat Knechtle

**Affiliations:** ^1^ Institute of Primary Care, University of Zurich, Zurich, Switzerland; ^2^ Ultra Sports Science Foundation, Pierre-Benite, France; ^3^ Departamento de Fisiologia, Disciplina de Neurofisiologia e Fisiologia do Exercício, Universidade Federal de São Paulo, São Paulo, Brasil; ^4^ Klinik für Allgemeine Innere Medizin, Kantonsspital St. Gallen, St. Gallen, Switzerland; ^5^ Centre of Research, Education, Innovation and Intervention in Sport (CIFI2D), Faculty of Sport, University of Porto, Porto, Portugal; ^6^ Medbase St. Gallen Am Vadianplatz, St. Gallen, Switzerland

**Keywords:** triathlon, split discipline, performance, prediction, race time

## Abstract

**Introduction:**

Our study examined 16,611 records of professional triathletes from 163 Ironman® 70.3 races across 97 countries (2004-2020). The aim was to identify the most predictive discipline—swim, bike, or run—for overall race time.

**Methods:**

We used correlation matrices to compare the dependent variable “finish time” with independent variables “swim time,” “bike time,” and “run time.” This analysis was conducted separately for male and female athletes. Additionally, univariate and multiple linear regression models assessed the strength of these associations.

**Results:**

The results indicated that “bike time” had the strongest correlation with finish time (0.85), followed by “run time” (0.75 for females, 0.82 for males) and “swim time” (0.46 for females, 0.63 for males). Regression models confirmed “bike time” as the strongest predictor of overall race time (*R*² = 0.8), with “run time” and “swim time” being less predictive.

**Discussion:**

The study concludes that in Ironman 70.3 races, “bike time” is the most significant predictor of overall race performance for both sexes, suggesting a focus on cycling in training and competition strategies. It also highlights a smaller performance gap between genders in swimming than in cycling or running.

## Introduction

The Ironman® 70.3, also known as the half-distance Ironman® triathlon, involves a 1.9 km swim, 90 km cycle, and 21.1 km run (1). It has been gaining popularity, as evidenced by an increase in both the number of races and competitors in recent years (1–3). This surge in interest has prompted a rise in scientific research focused on this triathlon distance. Recent studies have explored various factors related to Ironman® 70.3, including the impact of age (1, 2, 4, 5), sex differences in performance (1, 5), prior experience (4), and skeletal muscle damage during the race (6, 7).

Pacing is a crucial element in triathlon, providing valuable insights into which discipline is the strongest predictor of overall race performance (8–10). Research has primarily focused on other triathlon distances, such as the Sprint distance (750 m swim, 20 km bike, and 5 km run) (9, 11), the Olympic distance (1.5 km swim, 40 km bike, and 10 km run) (9, 12), and the full-distance Ironman® triathlon (3.8 km swim, 180 km bike, and 42.195 km run) (9, 13). According to studies, the predictive power of each split discipline varies depending on the length of the race (14). In the Sprint distance, the cycling split was the most predictive (9, 11), while in the Olympic distance, the swimming split was the most predictive (9). The cycling split proved most predictive for the Ironman® 70.3 (9), and in the full-distance Ironman® triathlon, the cycling split emerged as the strongest predictor (13, 15).

To date, only one study has compared various distances and found cycling to be the most important split discipline among professional Ironman® 70.3 triathletes (9). However, the study only considered a sample size of 5,258 male and 3,082 female professional triathletes, resulting in 8,340 records who competed in a 5-year period between 2015 and 2020 (9). Therefore, confirming this finding requires a larger sample size.

The aim of our study is to extend our understanding of the most predictive split discipline in Ironman® 70.3 by analyzing 16,611 records of professional triathletes who participated in 787 Ironman® 70.3 races between 2004 and 2020. Building on prior knowledge, we hypothesized that cycling would emerge as the most critical split discipline among elite Ironman® 70.3 triathletes in a vast sample size.

## Materials and methods

### Ethical approval

The ethikkommission kanton St. Gallen, Switzerland, approved this study (EKSG 01/06/2010). Since the study involved the analysis of publicly available data, the requirement for informed consent was waived.

### Data set and data preparation

The athletes' information was gathered from the official Ironman® website (https://ironman.com) utilizing a Python script. This provided access to athletes' gender, age, country of origin, overall race time, as well as split times for swimming, cycling, and running, as well as transitions 1 and 2, for all successful finishers of Ironman® 70.3 races recorded on the website between 2004 and 2020. For statistical analysis, we only considered data related to the times for each discipline, namely swimming (Swim Time), cycling (Bike Time), running (Run Time), and transitions (represented by transition 1—T1T, swimming to cycling, and transition 2—T2T, cycling to running) as well as overall race time (Finish Time). Only professional triathletes (https://www.ironman.com/pro-athletes) were included from each Ironman® 70.3 race, with exclusion criteria being: (i) athletes who did not start or finish; (ii) disqualified athletes; (iii) athletes with at least one missing split time; and (iv) inconsistent times (i.e., impossible split times or final times smaller than split times, etc.).

### Statistical analysis

The data distribution normality was checked by plotting the histograms of each split discipline and overall race times. Descriptive characteristics of these distributions are also presented in tables as means and standard deviations, along with maximum and minimum values. Two-way ANOVA and Tukey *post-hoc* tests were applied to assess the statistical significance of the results, which were considered significant at *p *< 0.05. The Pearson correlation was performed in order to verify the association level between the overall race time and the split times (swimming, running, cycling, and the first and second transitions) separately for males and females. Correlation coefficient classification criteria with 0.0–0.3 as a negligible correlation, 0.3–0.5 as a low correlation, 0.5–0.7 as a moderate correlation, 0.7–0.9 as a high correlation and 0.9–1.0 as a very high correlation were used (16). The overall race time is the addition of the partial times. This is deterministic, but nevertheless, regressions can be built around the dataset. In order to further understand the relationships between the data variables, a multiple linear regression (MLR) was built to model the relationship between split and overall race times with scatter plots to illustrate these associations.

The MLR model can be represented as follows:

Finish Time = f(ST, BT, RT, T1T, T2T) = K + a*ST + b*BT + c*RT + d*T1T + e*T2T

where:

ST – Swim Time

BT – Bike Time

RT – Run Time

T1T and T2T are transition times and a, b, c, d, e are the regression coefficients and K the intercept constant.

Also, three univariate linear regression (ULR) models were built to try and find the “most predictive” split discipline.

Finish Time (Swim Time) – S + s*ST

Finish Time (Bike Time) – B + b*BT

Finish Time (Run Time) – R + r*RT

where:

S, B and R are the intercept constants of each model

s, b and r are the regression coefficients of each model

ST, BT and RT are the Swim Time, Bike Time, and Run Time, respectively

All statistical analysis were performed using the language Python and Google Colab notebooks.

## Results

The complete dataset encompassed 787 Ironman® 70.3 competitions held in 197 distinct locations between 2004 and 2020, with a total of 840,075 successful finishers' records. The professional (PRO) group comprised 16,611 records, with 10,282 male and 6,329 female Ironman® 70.3 triathletes records from 97 countries competing in 163 unique events.

**Figure 1:**
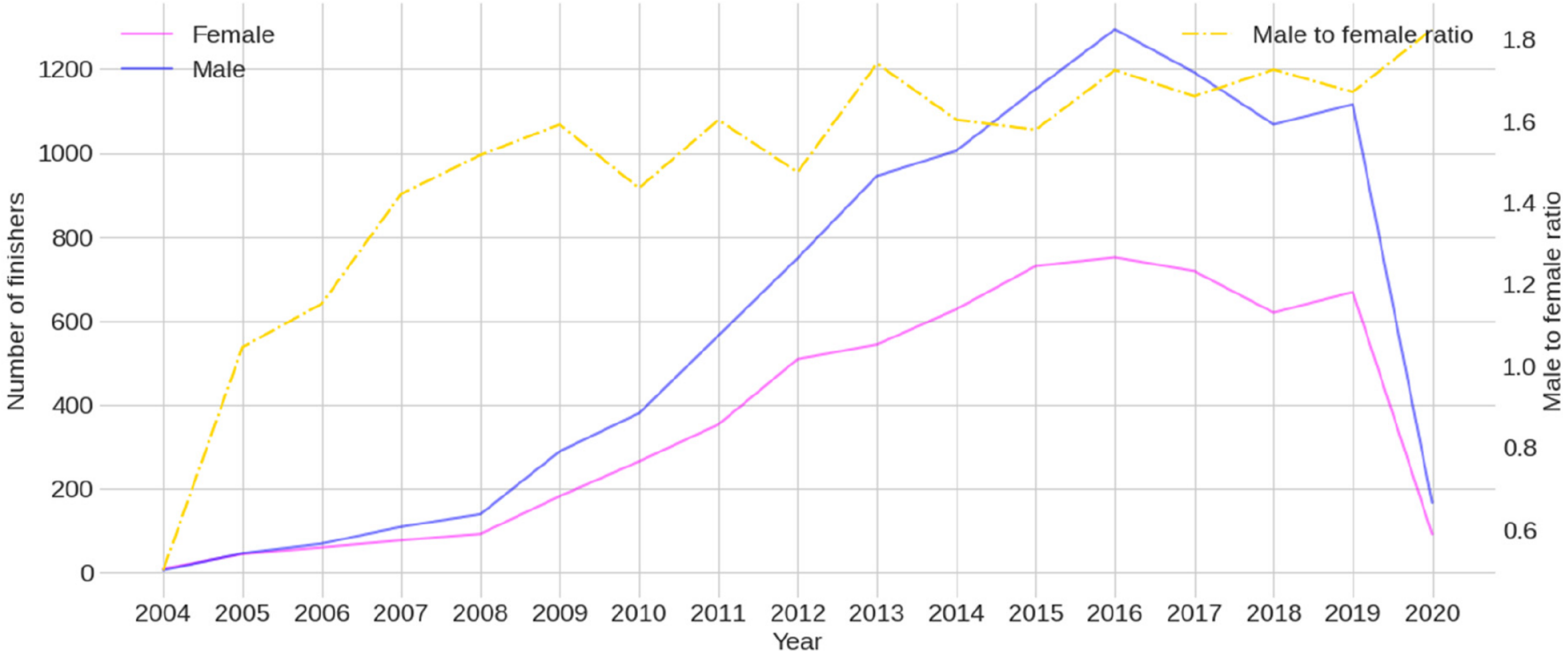
Trends in professional (PRO) female and male participation across years with the male-to-female ratio.

**Figure 2:**
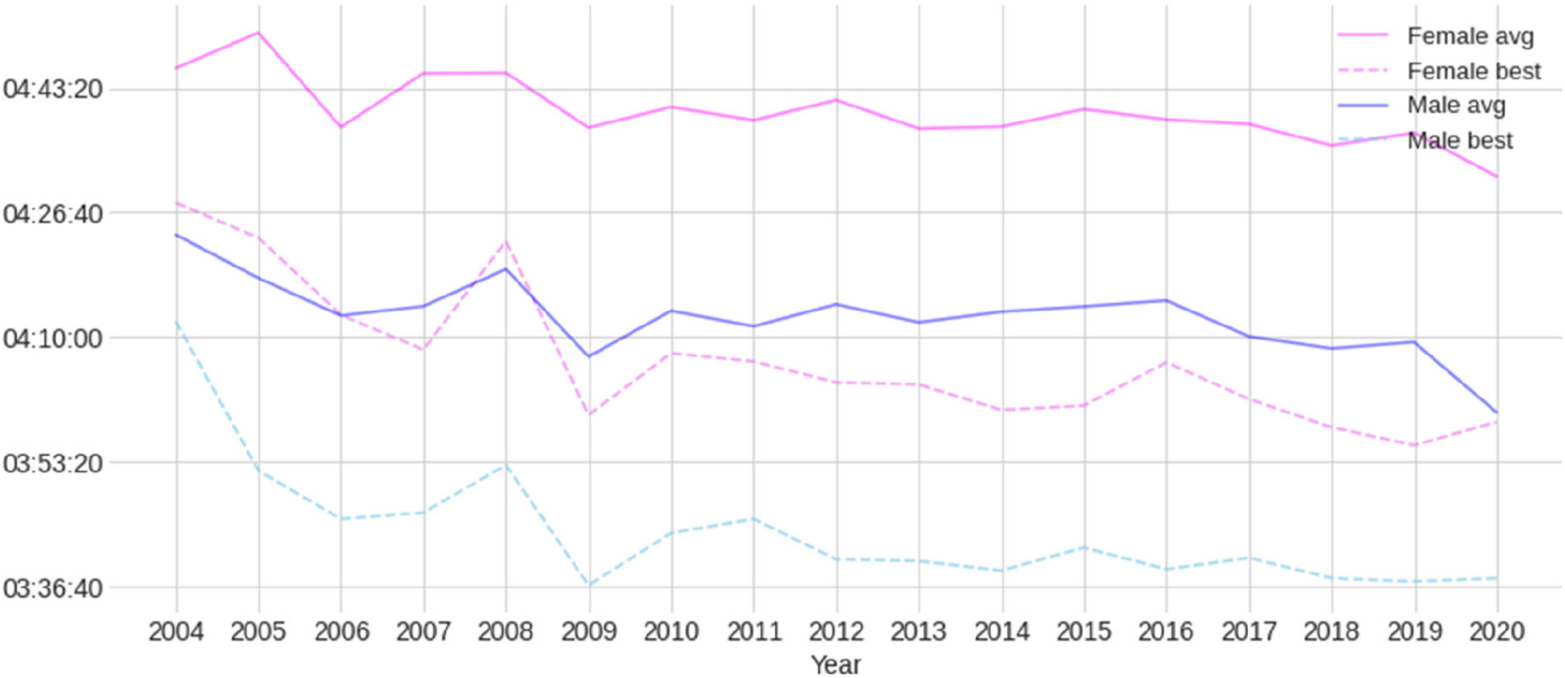
Trends in average and best overall race times for both professional (PRO) female and male athletes.

**Figure 3:**
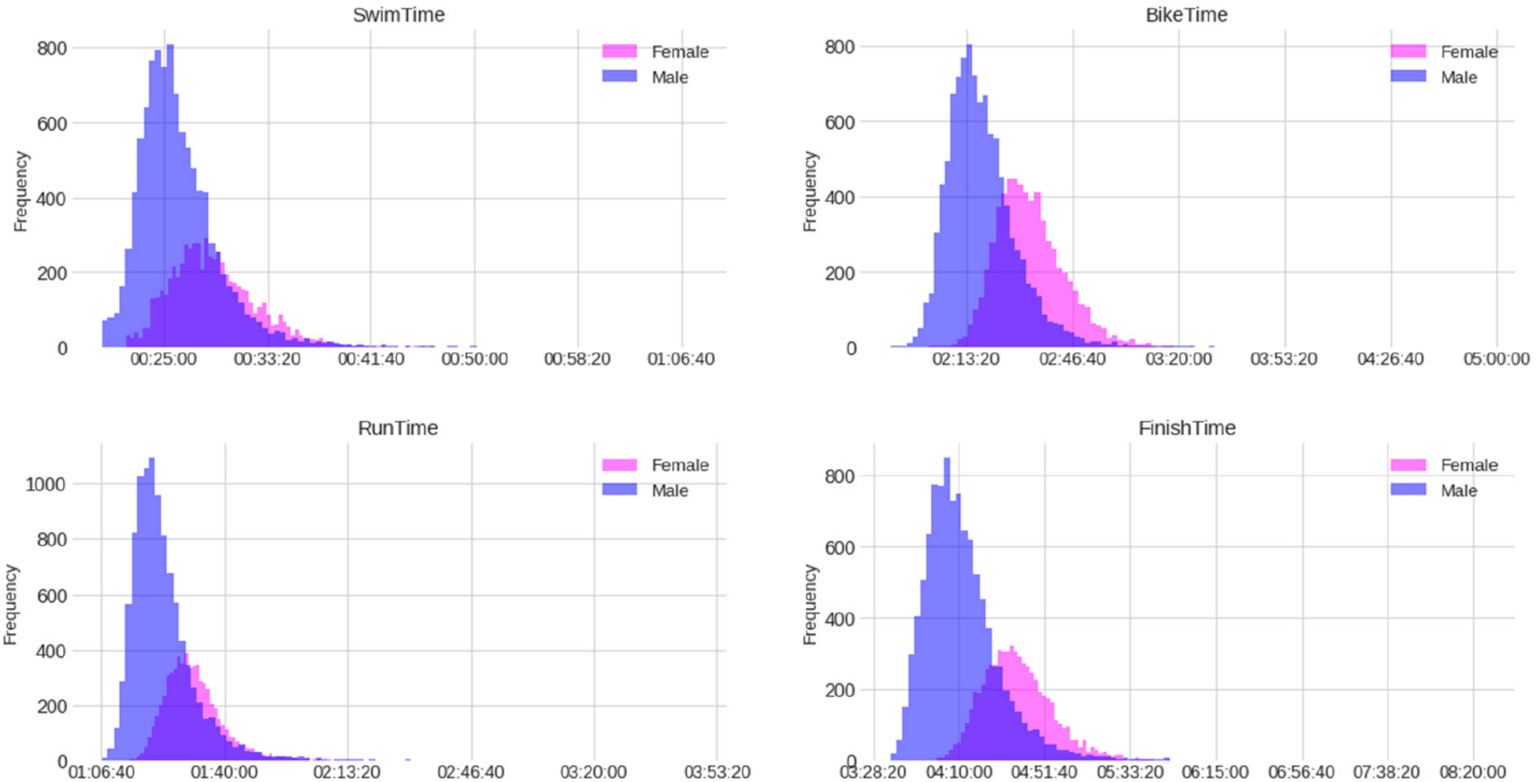
Distribution (histograms) of finish and split times by sex of the female and male professional (PRO) triathletes (times in HH:MM:SS).

Figure 1 presents the male and female participation trend in the PRO category and the male-to-female ratio between 2004 and 2020. The popularity of both sexes increased over the years, but in 2020, due to the COVID-19 pandemic, there was a significant reduction in participation. Figure 2 presents the trends in average and best overall race times for male and female athletes, showing a noticeable improvement in performance over the years. Basic descriptive statistics of the split times and overall race times can be found in Table 1, while Figure 3 shows the histograms representing the normality of their distributions.

**Table 1:** Finish and split times by sex of the professional triathletes. Times are presented in HH:MM:SS.

Male athletes				
	mean	std	max	min
Swim time	00:26:14	00:03:29	01:07:51	00:20:01
Transition 1 time	00:02:14	00:00:47	00:08:02	00:00:46
Bike time	02:18:00	00:11:06	04:39:01	01:49:25
Transition 2 time	00:01:43	00:00:45	00:08:15	00:00:46
Run time	01:23:39	00:10:14	03:47:40	01:06:50
Finish time	04:11:51	00:20:38	08:25:46	03:36:44
Female athletes				
	mean	std	max	min
Swim time	00:29:02	00:03:28	00:54:59	00:22:01
Transition 1 time	00:02:26	00:00:49	00:07:13	00:00:46
Bike time	02:33:32	00:11:10	04:56:15	02:01:30
Transition 2 time	00:01:47	00:00:42	00:07:56	00:00:46
Run time	01:32:02	00:08:33	02:51:31	01:12:21
Finish time	04:38:51	00:17:53	07:11:15	03:55:29

**Figure 4:**
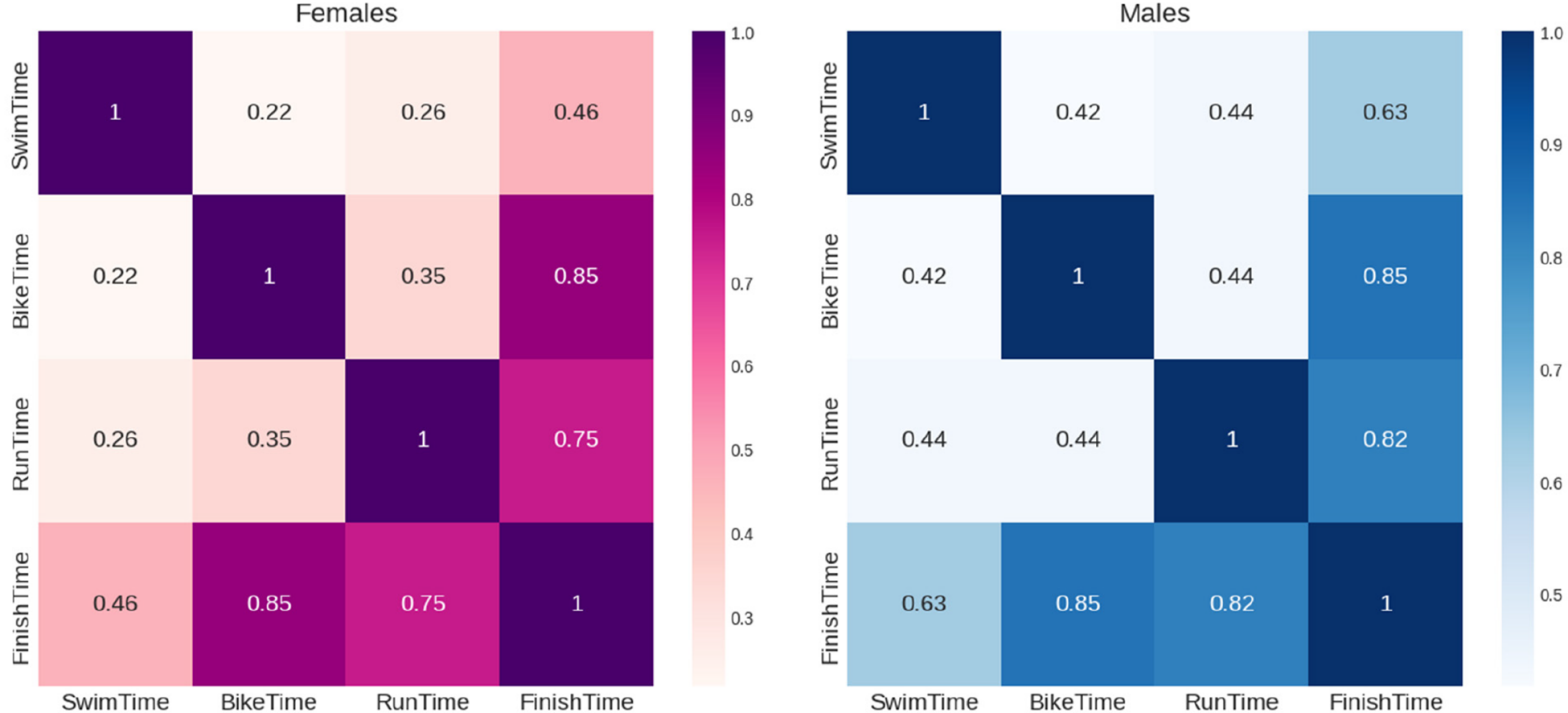
Pearson correlation matrix of finish and split times of the female and male professional triathletes.

According to the correlation analysis presented in Figure 4, the dependent variable “Finish Time” has a stronger association with the variables “Bike Time” and “Run Time” than with “Swim Time”. This trend is observed in both males and females, with the correlation with “Bike Time” being slightly higher than with “Run Time”. In absolute terms, high correlation coefficients were found for cycling in both males and females (*r* = 0.85). Similarly, a high correlation was observed for running in both females (*r* = 0.75) and males (*r* = 0.82). On the other hand, the correlation for swimming was low in females (*r* = 0.46) and moderate in males (*r* = 0.63).

**Figure 5:**
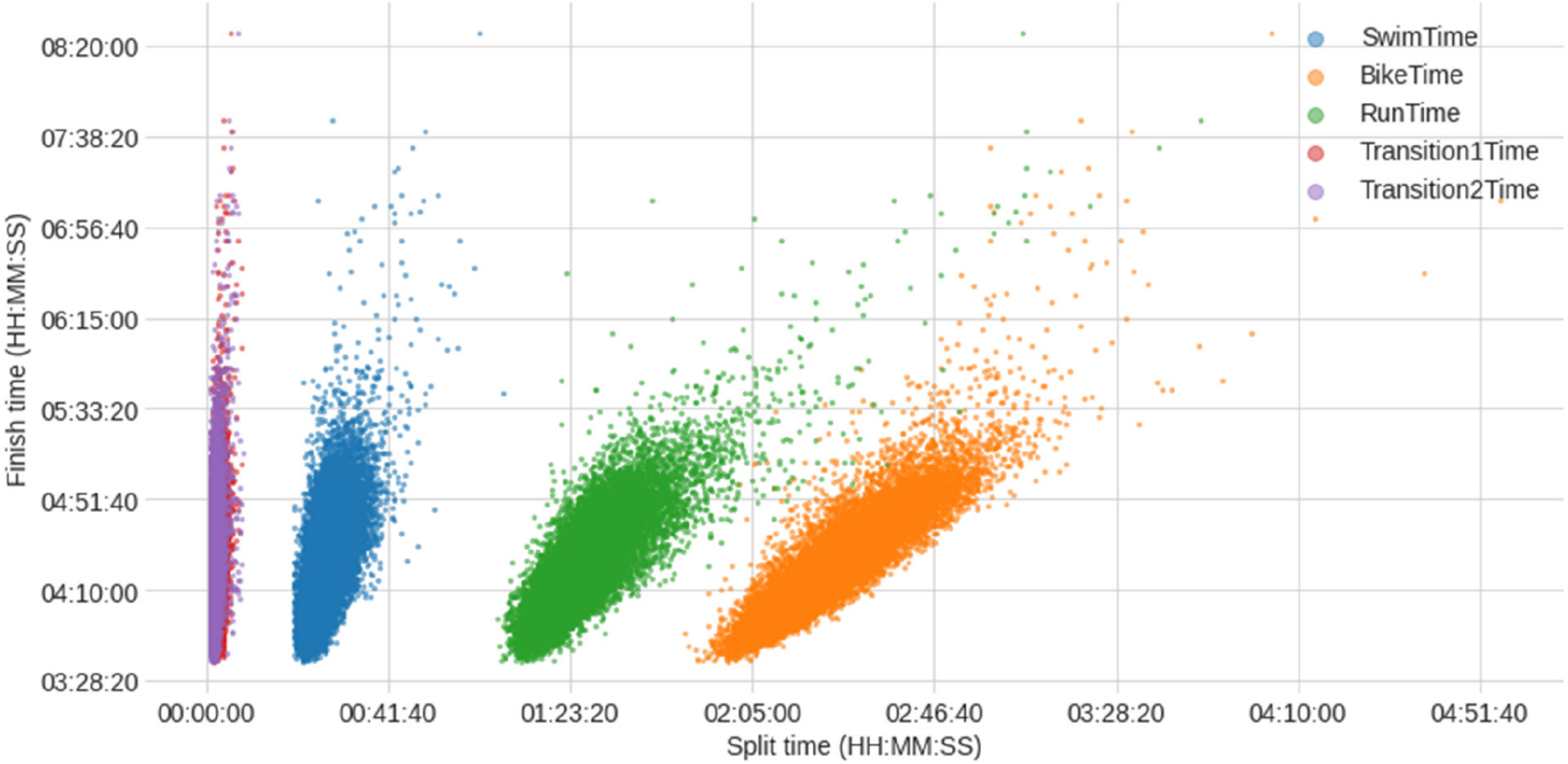
Scatter plots of finish and split times of the female and male professional (PRO) triathletes (times in HH:MM:SS).

Figure 5 presents the scatter plots of split times and overall race times. The scatter plots indicate a linear relationship between “Finish Time” and each split time. It also reveals the collinearity between “Run Time” and “Bike Time”. The “Finish Time” vs. “Run Time” (green) and “Finish Time” vs. “Bike Time” (orange) scatter plots show similar slopes. However, the “Finish Time” vs. “Swim Time” scatter plot (blue) may give the impression of a steeper slope, implying that losing a second during swimming significantly impacts overall time than during running or cycling. This is an illusion resulting from plotting all split times in the same chart. While swimming split times are shorter, cycling and running split times are longer, and hence contribute more to overall time. However, every second lost in any split discipline counts the same towards the overall finish time.

**Figure 6:**
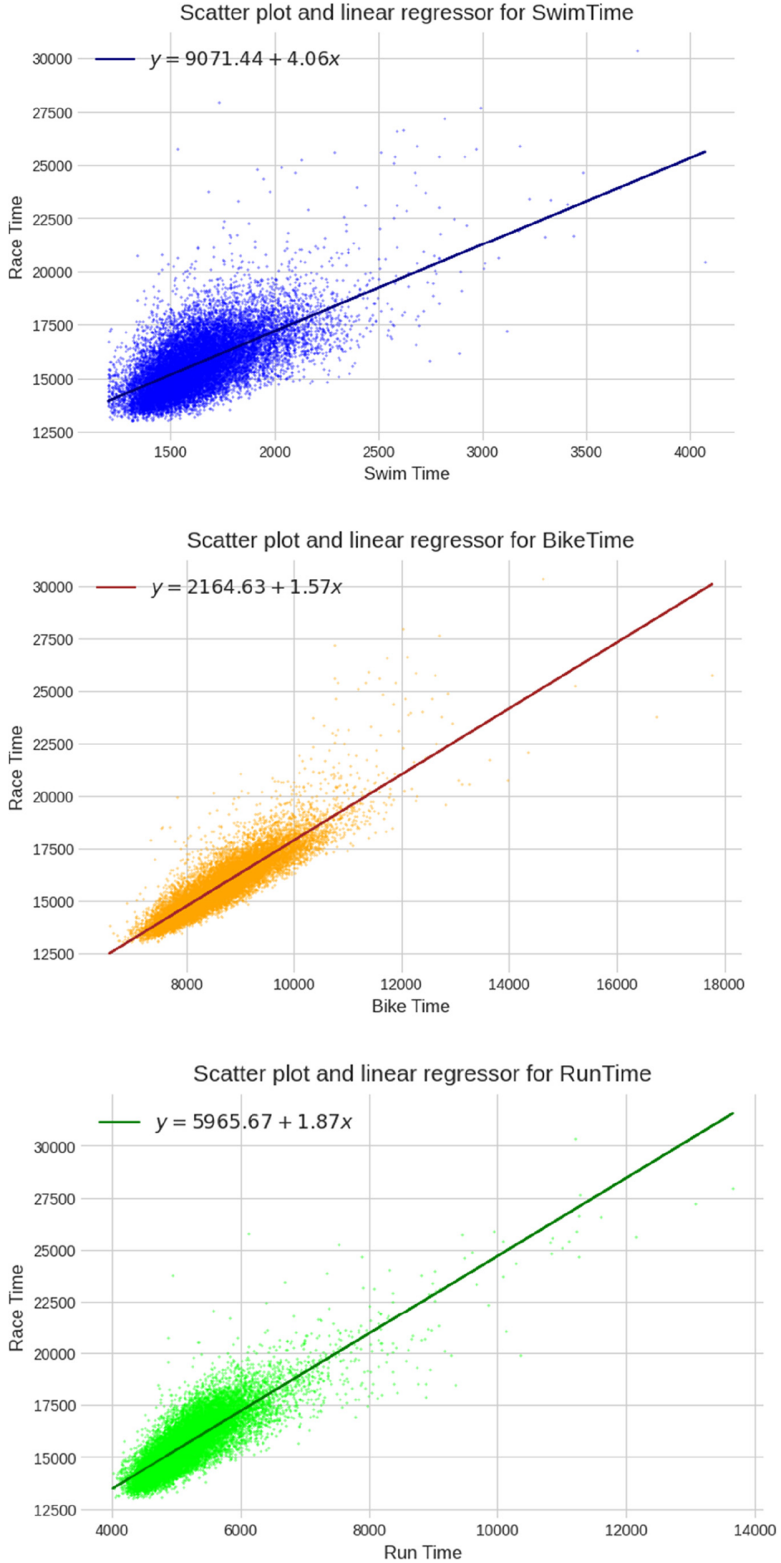
Univariate linear regressions for swimming, cycling and running (times in seconds) for both sexes together.

**Figure 7:**
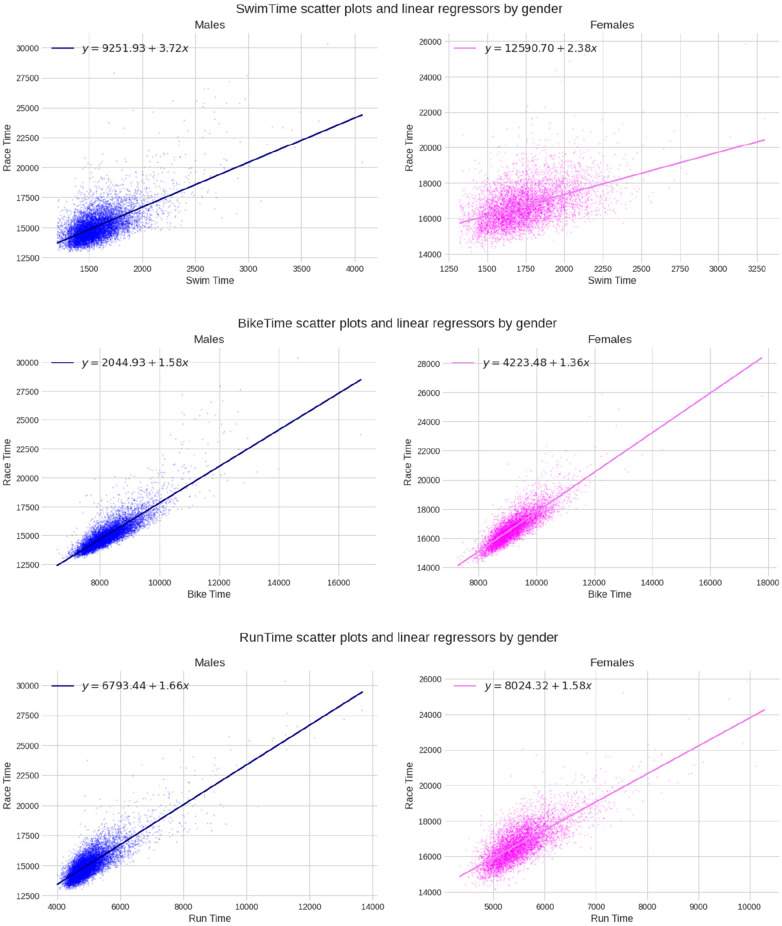
Univariate linear regressions for swimming, cycling, and running (times in seconds) for each sex.

The MLR model developed from the data yielded a perfect R^2^ score, indicating that the predictive model can accurately forecast the finish time based on the split disciplines (Table 2). The R2 values for the univariate regressors were 0.800 for cycling, 0.689 for running, and 0.414 for swimming, demonstrating the degree of contribution each split discipline has on the overall result. The scatter plots and univariate linear regressions for each split discipline are displayed in Figure 6 (both sexes) and Figure 7 (separated sexes), and cycling times performed best in the univariate model.

**Table 2:** Multiple linear regression of the female and male professional triathletes.

Dependent variable		Finish time				
Model		OLS (Ordinary least squares)				
R-squared		1.000				
	Coef	Std err	T	*P* > |t|	[0.025]	[0.975]
Const	−0.0765	0.027	−2.845	0.004	−0.129	−0.024
Swim time	0.9999	1.25e-05	7.99e + 04	<0.001	1.000	1.000
Bike time	1.0000	3.69e-06	2.71e + 05	<0.001	1.000	1.000
Run time	1.0000	4.67e-06	2.14e + 05	<0.001	1.000	1.000
Transition 1 time	0.9998	4.67e-06	1.83e + 04	<0.001	1.000	1.000
Transition 2 time	1.0003	6.03e-05	1.66e + 04	<0.001	1.000	1.000

**Figure 8:**
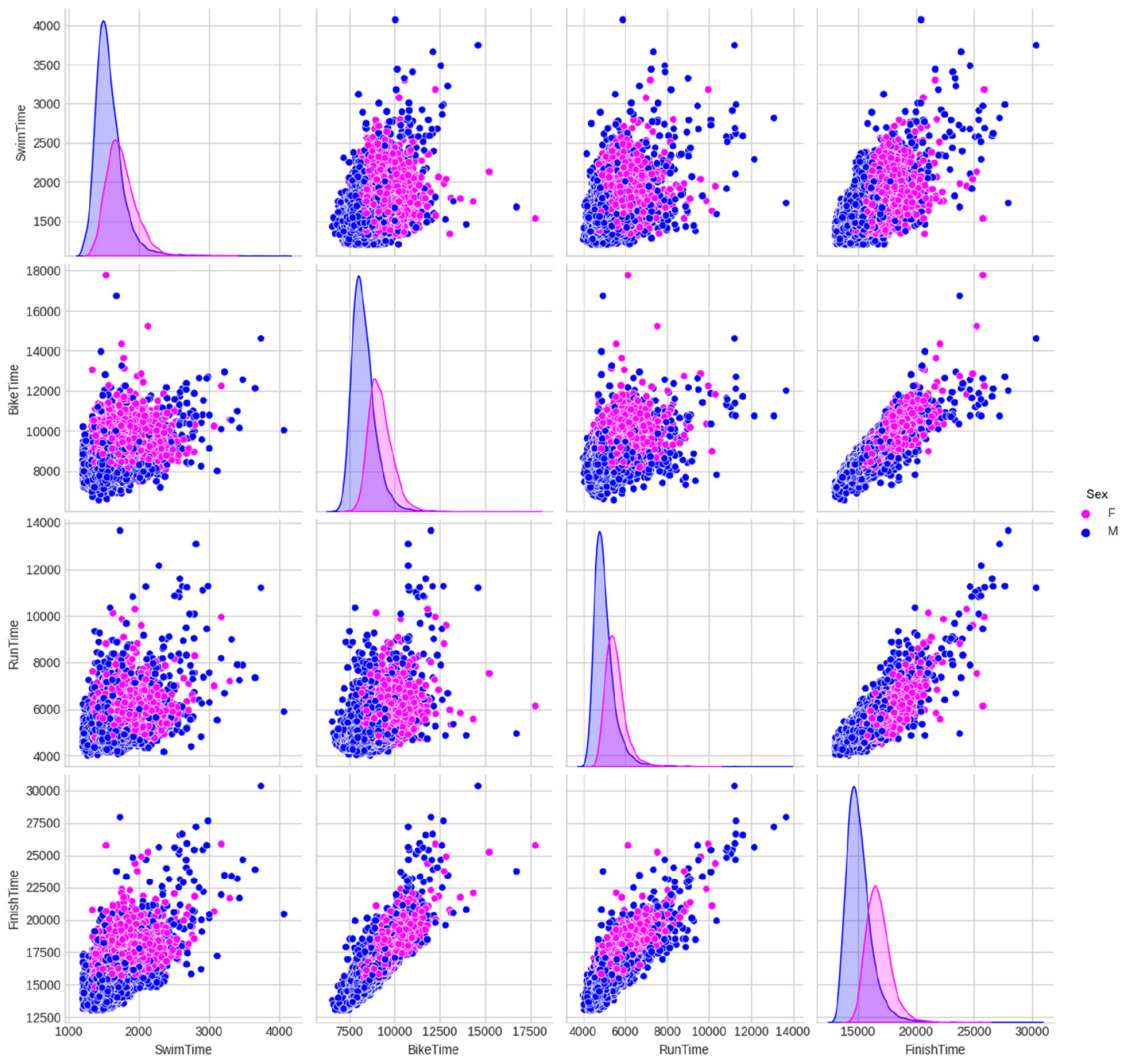
Relationships between split times of the female (F) and male (M) professional (PRO) triathletes by sex (scatter plots) along with the distributions of each variable in the diagonal (times in seconds).

Figure 8 presents a comprehensive view of the distribution of split times and overall race times, scatter plots among them, and the relationship between split times by sex (scatter plots) along with the variables' distributions in the diagonal. It is noteworthy that the male/female distributions of swim times are much closer when compared to the bike or run times, as previously observed when analyzing correlations. This suggests that male and female swimming performances were more similar than their running or cycling counterparts.

## Discussion

This study aimed to investigate how the various split disciplines impact the overall race times of elite Ironman® 70.3 triathletes, with a focus on determining which split discipline has the most significant effect. Our results support our hypothesis that cycling is the most critical discipline in Ironman® 70.3, contributing the most to the overall result and displaying more differences between sexes.

According to the correlation analysis, the overall race time was found to have a stronger association with cycling and running times compared to swimming times. Variability in running performance may be determined by differences in neuromuscular fatigue, as evidenced by decreases in peak power, peak velocity, and force rate during the triathlon event (17). The present study focused on professional athletes, yet a high degree of performance variability was observed among them, including minimum and maximum values for both finish and split times, which could be attributed to possible different competitive levels among the athletes. Moreover, intense cycling before running may impair running performance in terms of the kinematic, physiological, and perceptual index (18), which can negatively impact triathletes with low competitive levels and less experience in the sport (19).

A previous study investigating performance patterns in Olympic distance triathlon found similar results in terms of the association between running and overall time (8). The authors identified running as a critical predictive factor for overall race time, particularly for female athletes. Among female athletes ranked between 1st and 3rd positions, 46% recorded a time 0–10 s faster than the fastest split time in the run. For those ranked between 4th and 10th positions, approximately 95% recorded a time of 50 s or slower than the fastest split time in the run (8). Comparable results were noted among elite athletes competing in the Olympic distance, where the running split emerged as the predominant factor affecting overall race time, irrespective of position or sex (12). However, caution should be taken in generalizing and comparing these results, given methodological differences between studies, including variations in event characteristics, sample size, athlete performance level, age, sex, previous experience, temporal interval, and statistical approach (11, 20). Standardization of the outcome, as previously utilized, is therefore essential**.**

According to this study, swimming had the least impact on Ironman® 70.3 performance. In Olympic distance triathlons, research has demonstrated that a slower swimming performance can significantly improve subsequent cycling and overall race performance (21). To conserve energy but still maintain a position in the **“**first pack,**”** a previous review emphasized reducing the swimming intensity (22). Being in the first pack after the swim and running fast after cycling is crucial for achieving a top ranking in an Olympic distance triathlon (23). However, opting for a slower swim in this type of triathlon may be a strategy for cycling faster (24). Additionally, other studies have reported a weak correlation between swimming and overall finishing time (25, 26).

The order in which the three split disciplines are performed is crucial for the overall outcome in a triathlon race (27, 28). The efficiency of movement during swimming, cycling, and running is also closely related to overall race performance (29). Pacing during a triathlon race is particularly important and depends on both the distance and discipline (14). In addition to the split disciplines, the transitions between them are also crucial, particularly in relation to race length (28). In an Olympic distance triathlon, the running speed during transition 2 (from cycling to running) is pivotal for improving the run split (30). Similar results were found in a study of transitions in the Olympic Triathlon, where spending less time in transition 2 was linked to a better final result (25). Therefore, training plans for athletes competing in Ironman® 70.3 should focus on the disciplines that can significantly impact performance, considering the strengths and weaknesses of the athletes**.**

### Limitations

One limitation of this analysis is that training was not considered. The amount of training an athlete undergoes has a significant impact on race performance (4, 31–33). Additionally, physiological variables (31, 34–37), anthropometric characteristics (31, 34, 38), age (2, 4, 39), previous experience (4), and motivation (4, 38, 40) were not considered in this study. Moreover, the absence of information regarding ranking positions (such as top 3 or top 10) could hinder the generalization of results to a broader group. Furthermore, the original dataset was obtained from a public source and was non-consistent and incomplete. Even after all the procedures to clean up the dataset, there is a possibility that erroneous records were included in the analysis. The study's findings may be constrained by inherent limitations arising from the diverse route profiles in triathlon disciplines, encompassing running and cycling, as well as the influence of variable weather conditions. These factors introduce potential sources of error that could impact the accuracy and generalizability of the research outcomes**.**

## Conclusion

This study found that for both male and female professional finishers, cycling times were more predictive of overall race times than running and swimming times. In males, running and swimming times were more closely related to overall race times than in females. Swimming performances showed less disparity between sexes than running or cycling performances. These findings can guide athletes and coaches to better allocate their training efforts, emphasizing the disciplines impacting their race outcomes. This nuanced understanding of the relationship between discipline-specific times and overall race performance empowers triathletes to make informed decisions, optimize their training regimens, and ultimately enhance their competitiveness in the sport.

## Data Availability

The raw data supporting the conclusions of this article will be made available by the authors, without undue reservation.
